# Antitumoral Efficacy of the Protease Inhibitor Gabexate Mesilate in Colon Cancer Cells Harbouring KRAS, BRAF and PIK3CA Mutations

**DOI:** 10.1371/journal.pone.0041347

**Published:** 2012-07-24

**Authors:** Giovanni Brandi, Simona Tavolari, Francesco De Rosa, Stefania Di Girolamo, Valentina Agostini, Maria Aurelia Barbera, Giorgio Frega, Guido Biasco

**Affiliations:** 1 “L. and A. Seràgnoli” Department of Hematology and Oncological Sciences, Sant’Orsola-Malpighi Hospital, University of Bologna, Bologna, Italy; 2 “G. Prodi” Interdepartmental Center for Cancer Research (C.I.R.C.), University of Bologna, Bologna, Italy; 3 Center for Applied Biomedical Research (C.R.B.A.), Sant’Orsola-Malpighi Hospital, University of Bologna, Bologna, Italy; Ospedale Pediatrico Bambino Gesu’, Italy

## Abstract

The employment of anti-epidermal growth factor receptor (EGFR) antibodies represents a backbone of the therapeutic options for the treatment of metastatic colorectal cancer (mCRC). However, this therapy is poorly effective or ineffective in unselected patients. Mutations in KRAS, BRAF and PIK3CA genes have recently emerged as the best predictive factors of low/absent response to EGFR-targeted therapy. Due to the need for efficacious treatment options for mCRC patients bearing these mutations, in this short report we examined the antitumoral activity of the protease inhibitor gabexate mesilate, alone and in combination with the anti-EGFR monoclonal antibody cetuximab, in a panel of human CRC cell lines harbouring a different expression pattern of wild-type/mutated KRAS, BRAF and PIK3CA genes. Results obtained showed that gabexate mesilate significantly inhibited the growth, invasive potential and tumour-induced angiogenesis in all the CRC cells employed in this study (including those ones harbouring dual KRAS/PIK3CA or BRAF/PIK3CA mutation), while cetuximab affected these parameters only in CRC cells with KRAS, BRAF and PIK3CA wild-type. Notably, the antitumoral efficacy of gabexate mesilate and cetuximab in combination was found to be not superior than that observed with gabexate mesilate as single agent. Overall, these preliminary findings suggest that gabexate mesilate could represent a promising therapeutic option for mCRC patients, particularly for those harbouring KRAS, BRAF and PIK3CA mutations, either as mono-therapy or in addition to standard chemotherapy regimens. Further studies to better elucidate gabexate mesilate mechanism of action in CRC cells are therefore warranted.

## Introduction

In the last fifteen years, the introduction of at least six key drugs (oxaliplatin, irinotecan, capecitabine, bevacizumab, cetuximab and panitumumab), after the “era” of 5-fluorouracil as a single agent, has improved median overall survival of metastatic colorectal cancer (mCRC) patients up to 24 months [1], [2]. The employment of the anti-epidermal growth factor receptor (EGFR) antibodies panitumumab and cetuximab, either as mono-therapy or in addition to standard chemotherapy regimens, represents a backbone of the therapeutic options for the treatment of mCRC. However, randomized controlled trials have provided compelling evidence that EGFR-targeted therapy is poorly effective or ineffective in unselected mCRC patients. In recent years, activating mutations at codons 12 and 13 in the KRAS oncogene (KRAS^G12V^ and KRAS^G13D^) have emerged as the best predictive factors of low/absent response to anti-EGFR therapy in these patients, either in the first-line or subsequent lines of treatment [3]–[5]. For this reason, mCRC patients are now profiled for KRAS mutation and the employment of cetuximab and panitumumab is currently restricted only to those ones bearing the KRAS wild-type, as recommended by the European Medical Agency and the American Society of Clinical Oncology [6]. Although the presence of wild-type KRAS seems to be a condition for response to EGFR-targeted therapy, up to 50–65% of mCRC patients fail to benefit from this treatment, due to additional intrinsic resistance mechanisms [7]. In this regard, the involvement of BRAF^V600E^ and PIK3CA^H1074R^ at exon 20 mutations in the failure of such therapy has recently emerged [7]–[11]. Due to the lack of an effective targeted therapy, the discovery of new therapeutic options for mCRC patients with mutated KRAS, BRAF and PIK3CA genes represents therefore an intense area of investigation.

The protease inhibitor gabexate mesilate has been shown to exert a significant antitumoral activity in CRC cells, both in vitro and in vivo [12]. However, the effect of this drug, alone or in combination with cetuximab, in human CRC cells harbouring a different expression pattern of wild-type/mutated KRAS, BRAF and PIK3CA still remains unsettled. The present study aims at investigating this hypothesis.

## Results

We preliminary selected a panel of human CRC cells harbouring a different expression pattern of wild-type/mutated KRAS, BRAF and PIK3CA genes. To this purpose, based on the Catalogue of Somatic Mutations in Cancer (COSMIC) database (http://www.sanger.ac.uk/genetics/CGP/cosmic/) and on the study of Jhawer et al. [13], CACO-2, SW48, HT-29, Colo205, SW480, SW620, RKO, LS174T and HCT-116 CRC cells were chosen (see Table 1 for their corresponding KRAS, BRAF and PIK3CA status). Notably, no CRC cell line with co-occurring KRAS and BRAF mutations was found and included in the present study, according to previous reports showing a pattern of mutual exclusivity for KRAS and BRAF mutation in human CRC [14]. The effect of gabexate mesilate, alone and in combination with cetuximab, was then investigated on CACO-2, SW48, HT-29, Colo205, SW480, SW620, RKO, LS174T and HCT-116 cell growth, invasive potential and tumour-induced angiogenesis, as they represents three fundamental processes in cancer onset and progression.

**Table 1:** KRAS, BRAF and PIK3CA status of the CRC cell lines included in the study.

Cell line	KRAS	BRAF	PIK3CA
**CACO-2** *	wild-type	wild-type	wild-type
**SW48** *	wild-type	wild-type	wild-type
**HT-29** *	wild-type	mutation at exon 15 (V600E)	wild-type
**Colo205** *	wild-type	mutation at exon 15 (V600E)	wild-type
**SW480** **	mutation at exon 2 (G12V)	wild-type	wild-type
**SW620** *	mutation at exon 2 (G12V)	wild-type	wild-type
**RKO** *	wild-type	mutation at exon 15 (V600E)	mutation at exon 20 (H1074R)
**LS174T** *	mutation at exon 2 (G12V)	wild-type	mutation at exon 20 (H1074R)
**HCT-116** *	mutation at exon 2 (G13D)	wild-type	mutation at exon 20 (H1074R)

KRAS, BRAF and PIK3CA status of the CRC cell lines included in the study.

* from COSMIC database (http://www.sanger.ac.uk/genetics/CGP/cosmic/).

** KRAS/BRAF from COSMIC database; PIK3CA from Jhawer et al. 2008.

**Figure 1:**
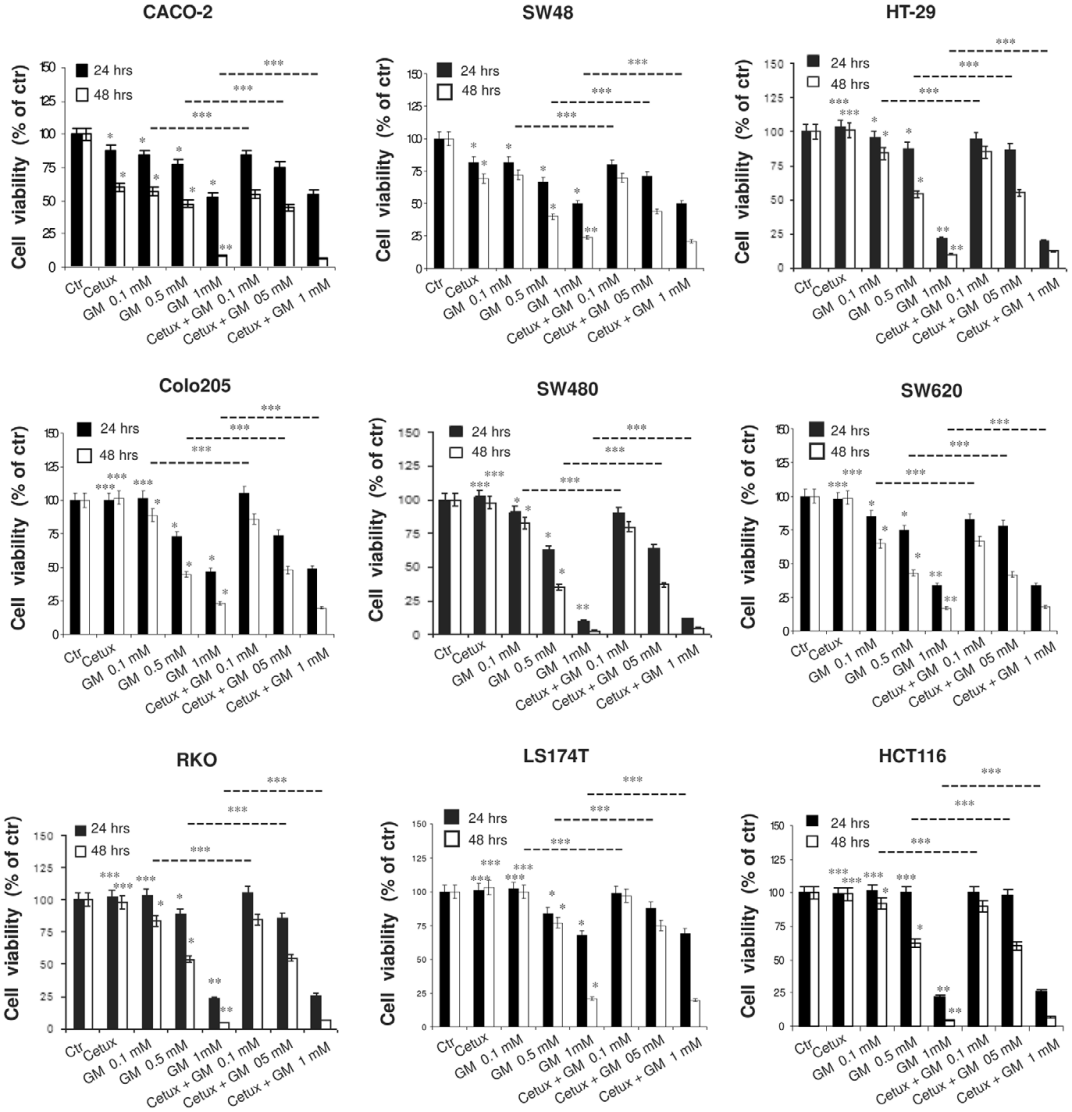
Effect of gabexate mesilate and cetuximab, alone and in combination, on CRC cell viability. Evaluation by MTT assay of CACO-2, SW48, HT-29, Colo205, SW480, SW620, RKO, LS174T and HCT-116 cell viability after 24 and 48 h of treatment with cetuximab 100 µg/ml and gabexate mesilate 0.1–1 mM, alone and in combination. Three independent experiments were performed. For each cell line, the mean value of untreated samples was assumed as 100% and mean values of treated cells were plotted as percentages with respect to their matched controls. Cetux: cetuximab; GM: gabexate mesilate. *p<0.05; **p<0.001; ***p : not significant.

As shown in Figure 1, 24 and 48 hrs of treatment with cetuximab (100 µg/ml) were found to affect cell viability only in CRC cells harbouring wild-type KRAS, BRAF and PIK3CA genes (CACO-2 and SW48), as expected. Conversely, gabexate mesilate (0.1–1 mM) induced a significant time and dose-dependent decrease of this parameter in all the CRC cell lines included in the study (CACO-2: IC_50_ 48 hrs  = 0.31±0.01; SW48: IC_50_ 48 hrs  = 0.33±0.01; HT-29: IC_50_ 48 hrs  = 0.55±0.03 mM; Colo205: IC_50_ 48 hrs  = 0.46±0.02; SW480: IC_50_ 48 hrs  = 0.45±0.02 mM; SW620: IC_50_ 48 hrs  = 0.39±0.02; RKO: IC_50_ 48 hrs  = 0.49±0.01 mM; LS174T: IC_50_ 48 hrs  = 0.59±0.03; HCT-116: IC_50_ 48 hrs  = 0.56±0.0 3 mM). We observed that the effect elicited on cell viability by gabexate mesilate alone was similar to that observed when this drug was administered in combination with cetuximab 100 µg/ml (Figure 1).

**Figure 2:**
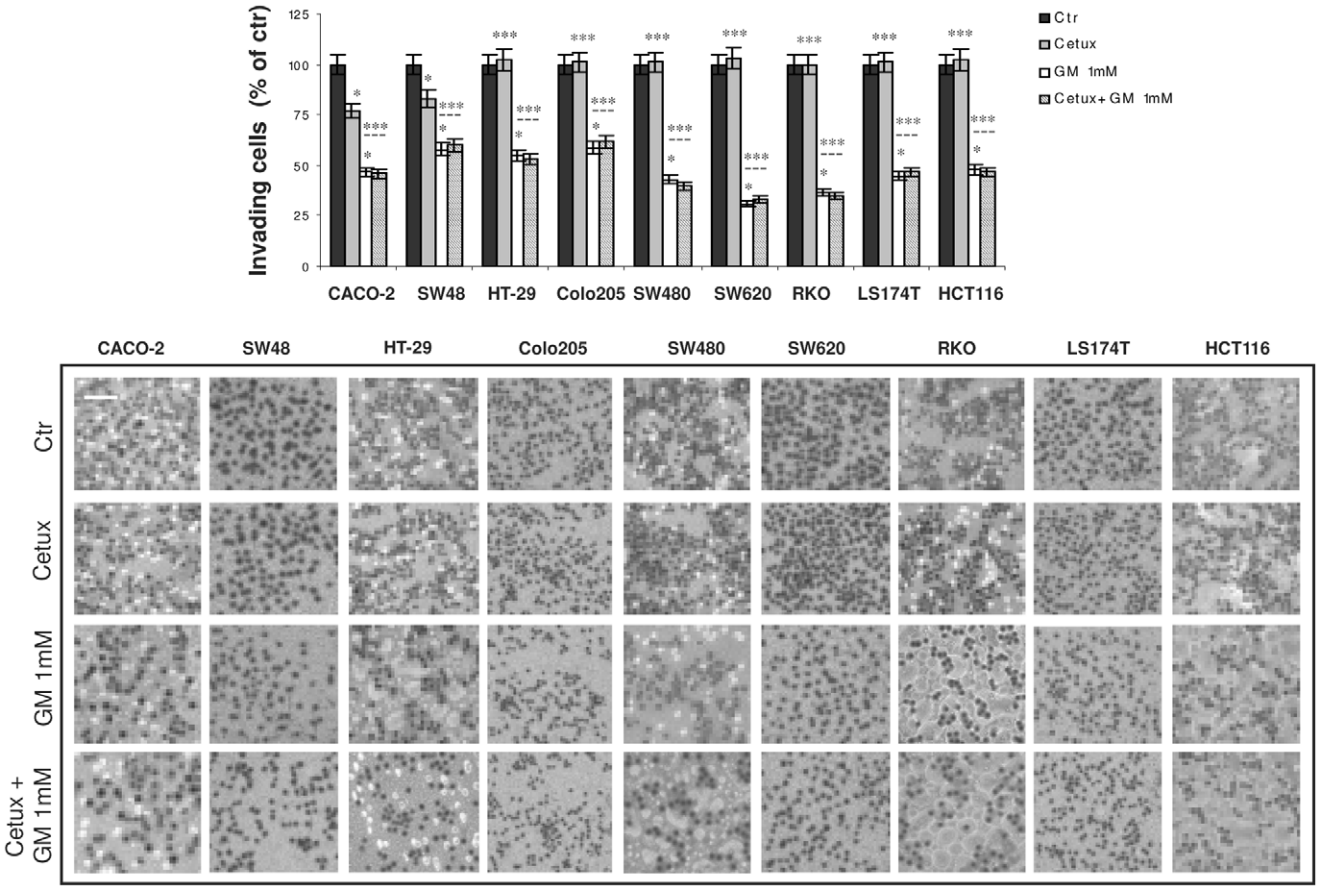
Effect of gabexate mesilate and cetuximab, alone and in combination, on CRC invasive potential. Evaluation by Boyden chamber invasion assay of CACO-2, SW48, HT-29, Colo205, SW480, SW620, RKO, LS174T and HCT-116 invasive potential after 6 hrs of treatment with cetuximab 100 µg/ml and gabexate mesilate 1 mM, alone and in combination. For each cell line, the mean value of untreated samples was assumed as 100% and mean values of treated cells were plotted as percentages with respect to their matched controls. Photographs of invading cells are representative of three independent experiments with similar findings. Cetux: cetuximab; GM: gabexate mesilate. Scale bar: 50 µm. *p<0.05; ***p : not significant.

Next, we investigated the effect of these two drugs on CACO-2, SW48, HT-29, Colo205, SW480, SW620, RKO, LS174T and HCT-116 invasiveness. Treatment for 6 hrs with gabexate mesilate 1 mM significantly decreased the invasive potential in all the CRC cell lines tested (CACO-2∶53%; SW48∶42%; HT-29∶45%; Colo205∶43%; SW480∶55%; SW620∶69%; RKO: 63%; LS174T: 54%; HCT-116∶52%) (Figure 2). Notably, such a decrease was not due to a cytotoxic effect of this drug, as confirmed by a parallel experiment in which no significant change of cell viability between control and treated cells was observed at this time of treatment (data not shown). Conversely, 6 hrs of treatment with cetuximab 100 µg/ml induced no significant changes on CRC cell invasive potential, except on CACO-2 and SW48 cells, where a 25% and 22% of inhibition was observed, respectively. Again, when this drug was employed in combination with gabexate mesilate, the effect elicited was similar to that observed with gabexate mesilate as single agent (Figure 2).

**Figure 3:**
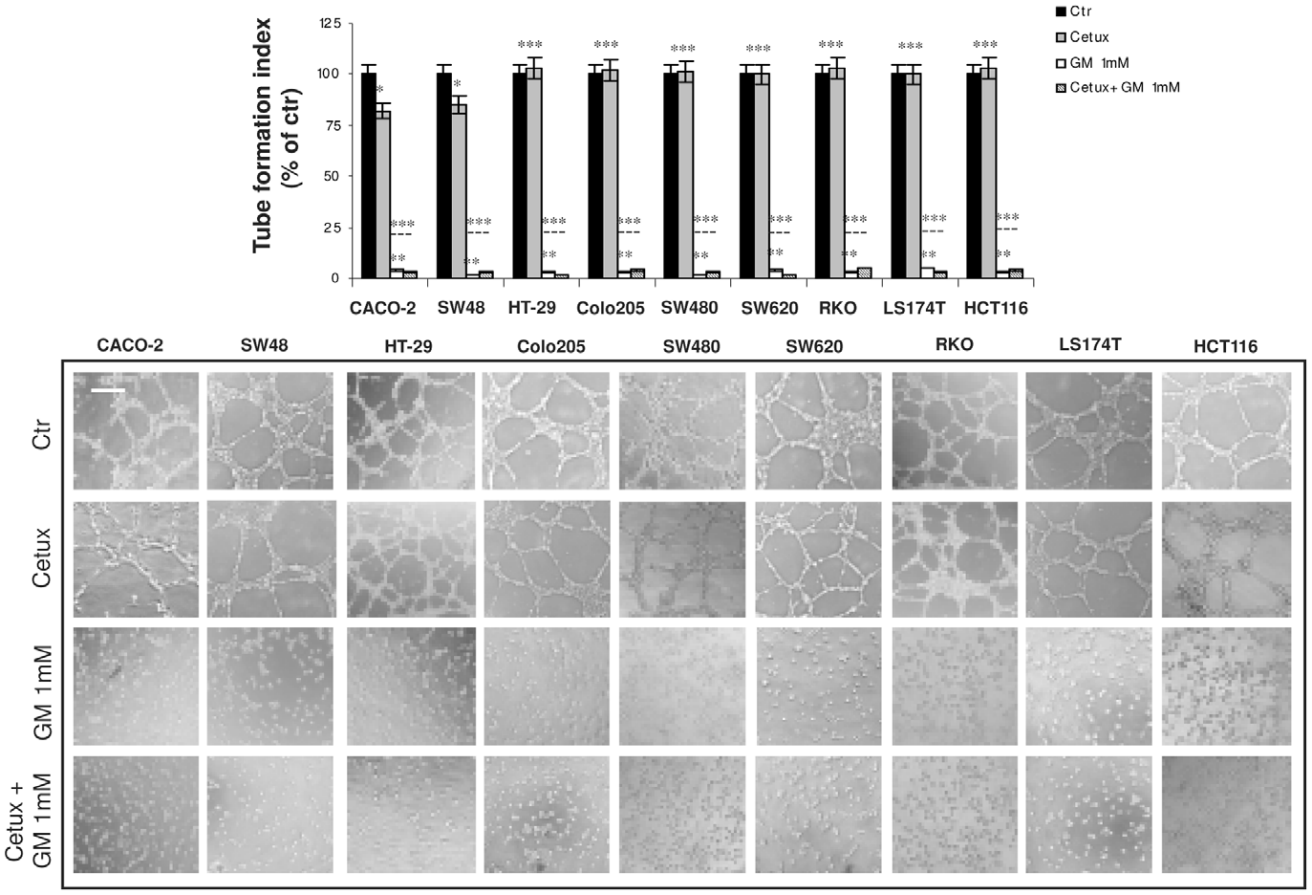
Effect of gabexate mesilate and cetuximab, alone and in combination, on CRC conditioned medium-induced angiogenesis. Evaluation by in vitro Matrigel angiogenesis assay of EA.hy926 endothelial cell differentiation in capillary-like structures after 24 hrs incubation with the conditioned medium of CACO-2, SW48, HT-29, Colo205, SW480, SW620, RKO, LS174T and HCT-116 cells, previously treated for 6 hrs with cetuximab 100 µg/ml and gabexate mesilate 1 mM, alone and in combination. Tube formation index was assessed as described in Material and Methods section. For each cell line, the mean value of untreated samples was assumed as 100% and mean values of treated cells were plotted as percentages with respect to their matched controls. Photographs are representative of three independent experiments with similar findings. Cetux: cetuximab; GM: gabexate mesilate. Scale bar: 50 µm. *p<0.05; **p<0.001; ***p : not significant.

Lastly, we assessed the effect of cetuximab and gabexate mesilate on tumour-induced angiogenesis. A very strong inhibitory effect on this parameter was observed when we examined the action of gabexate mesilate alone. Indeed, 24 hrs incubation of EA.hy926 cells with the conditioned medium of CACO-2, SW48, HT-29, Colo205, SW480, SW620, RKO, LS174T and HCT-116 cells, previously treated for 6 hrs with this drug at the dose of 1 mM, almost completely inhibited endothelial cell differentiation in capillary-like structures, as indicated by the presence of an interconnected network of anastomosing cells in control samples and of spherical cells, isolated or aggregated in small clumps, in treated ones (Figure 3). Also on this parameter, cetuximab alone (100 µg/ml) was found to be moderately effective only in CRC cell lines with wild-type KRAS, BRAF and PIK3CA genes (inhibition of tube formation index, 22% and 20% on CACO-2 and SW48 cells, respectively). Due to the strong inhibition of tumor-induced angiogenesis by gabexate mesilate alone at the dose 1 mM, the effect of this drug alone and in combination with cetuximab was also assessed at the dose of 0.1 and 0.5 mM. However, also at these doses, the anti-angiogenic effect displayed by gabexate mesilate plus cetuximab was not superior than that observed with gabexate mesilate employed as single agent (data not shown).

Overall these findings, besides confirming the lack of response to cetuximab in CRC cells with KRAS, BRAF and PIK3CA mutations, indicate that gabexate mesilate is able to exert a significant antitumoral activity in CRC cells harbouring either wild-type or mutated KRAS, BRAF and PIK3CA genes, with an efficacy comparable to that observed when it is used in combination with anti-EGFR antibodies.

## Discussion

Personalized therapy represents an attractive goal in oncology. To date, cancer genome sequencing has become a powerful tool to identify cancer-related mutations and to select patients who could benefit from a specific therapeutic regimen. In this context, analysis of KRAS mutation has rapidly increased the therapeutic index of EGFR-targeted therapy in mCRC patients, restricting this treatment only to those ones harbouring KRAS wild-type. Although further validation is required before a possible application in clinical practice, it has become clear that BRAF and PIK3CA additional genotyping could significantly improve the objective response rate in these patients. Indeed, activating KRAS, BRAF and PIK3CA mutations are able to bypass EGFR pharmacological inhibition, thereby resulting in a constitutive activation of the mitogen-activated protein kinase and AKT pathways, known to play a central role in cancer onset and progression [15]. In terms of therapeutic implications, this suggests that also mCRC patients with mutated BRAF and PIK3CA genes should gain little or no benefit from EGFR-targeted therapy. Notably, KRAS, BRAF and PIK3CA mutations are commonly activated (up to 50–60% as a whole) in CRC patients [14]. For this reason, the discovery of new treatment options effective in mCRC patients bearing such mutations currently represents an intensive area of investigation [11], [16]–[18].

The protease inhibitor gabexate mesilate is a drug routinely employed in clinical practice in Italy, Japan and Korea for the treatment of acute pancreatitis and disseminated intravascular coagulation, together with the prophylaxis of post-endoscopic retrograde cholangiopancreatography pancreatitis [19], [20]. Notably, gabexate mesilate toxicological profile has been tested in humans and severe side-effects have seldom been described. Interestingly, in previous preclinical studies, this drug demonstrated a potent antitumoural activity, including inhibition of CRC primary tumour growth and liver metastases in nude mice [12].

This preclinical study aimed to investigate the putative antitumoral efficacy of gabexate mesilate, alone and in combination with the anti-EGFR monoclonal antibody cetuximab, in a panel of human CRC cell lines harbouring a different expression pattern of wild-type/mutated KRAS, BRAF and PIK3CA genes. Results obtained (besides confirming the lack of response to cetuximab in CRC cells bearing such mutations) showed that gabexate mesilate was able to exert a broad antitumoral effect in such cells, being able to significantly affect the cell viability, invasive potential and tumour-induced angiogenesis in all the cell lines tested in this study, including those ones harbouring dual KRAS/PIK3CA or BRAF/PIK3CA mutation. The effectiveness of gabexate mesilate as single agent was found to be comparable to that observed when this drug was used in combination with cetuximab, thus indicating that the antitumoral efficacy of these drugs in combination was not superior than that of gabexate mesilate employed as single agent.

Notably, cell growth, a high invasive potential and tumor-induced angiogenesis are three pivotal processes for cancer development and metastatisation, thus representing an attractive target for cancer treatment. As a matter of fact, many therapeutic strategies based on the integration of selective inhibitors of these pathways are now being widely explored in preclinical and clinical studies. In this context, also taking into account the good toxicological profile, the preliminary results showed in this short report suggest that gabexate mesilate could represent a promising therapeutic option for mCRC patients, particularly for those ones bearing mutated KRAS, BRAF and PIK3CA genes, either as mono-therapy or in addition to standard chemotherapy regimens. Further studies to better elucidate gabexate mesilate mechanism of action in CRC cells are therefore warranted.

## Materials and Methods

### Cells and Cell Culture

The human CRC cell lines HT-29, LS174T and HCT-116, as well as the human immortalized endothelial-like EA.hy926 cell line, were obtained from American Type Culture Collection (ATCC, Manassas, VA, USA). RKO, CACO-2, SW480 and SW48, SW620 and Colo205 human CRC cell lines were a generous gift of Dr. Luigi Ricciardiello (Department of Clinical Medicine, Sant’Orsola-Malpighi Hospital, University of Bologna, Bologna, Italy) and Prof. Massimo Derenzini (Clinical Department of Radiological and Histopathological Sciences, Sant’Orsola-Malpighi Hospital, University of Bologna, Bologna, Italy), respectively. All cell lines were cultured in Dulbecco’s modified Eagle’s Medium with 4.5 g/L glucose (Euroclone, Milan, Italy), supplemented with 10% (v/v) heat-inactivated FBS (Euroclone), 2 mM L-glutamine, 100 U/ml penicillin and 100 µg/ml streptomycin (Sigma-Aldrich, St. Louis, MO, USA). Cells were grown at 37°C in a humidified atmosphere of 95% air and 5% CO_2_ and routinely passaged using trypsin-EDTA 0.025% (Sigma-Aldrich). For each cell line employed in this study, a cell line authentication test by evaluating DNA short tandem repeat (STR) profile was performed, in order to exclude a contamination or misidentification with other cell lines.

### Drug Treatments

Gabexate mesilate (Foy, Sanofi Aventis, Milan, Italy) and cetuximab (Erbitux, (Merck, Darmstadt, Germany) were diluted into the medium to obtain the required final concentration before each experiment. For both drugs, the doses employed (100 µg/ml for cetuximab and 0.1–1 mM for gabexate mesilate) were chosen according to previous in vitro studies [12], [13], [21], [22]. As gabexate mesilate has been reported to be partially degraded by serum albumin [23], all experiments were performed in serum-free medium. In experiments conducted with the conditioned medium of CRC cell lines, cells were treated with drugs, alone and in combination, for 6 hrs and then conditioned medium was harvested, centrifuged at 500×g for 5 min at 4°C to remove cells and debris, and frozen at −80°C until use.

### Cell Viability Assay

Cell viability experiments were carried out by 3-[4,5-dimethylthiazol-2-yl]-2,5-diphenyltetrazolium bromide (MTT) assay. Briefly, CACO-2, SW48, HT-29, Colo205, SW480, SW620, RKO, LS174T and HCT-116 cells (1×10^4^ cells/well) were plated in a 96-well plate in quadruplicate and allowed to adhere for 24 hrs. Then cells were treated with cetuximab and increasing concentrations of gabexate mesilate for 24 and 48 hrs. At the end of incubation, MTT was added to each well and cells incubated at 37°C for 4 hrs. Formazan crystals were then dissolved by DMSO addition. Absorbance was then measured at 570 nm in a 96-well spectrophotometric microplates reader (Bio-Rad, Hercules, CA, USA). For each cell line, the IC_50_ value was calculated by nonlinear regression analysis using PRISM 5.01 software version (GraphPad Software, San Diego, CA, USA).

### Chemoinvasion Assay

The invasive potential of CACO-2, SW48, HT-29, Colo205, SW480, SW620, RKO, LS174T and HCT-116 cells was assessed by chemoinvasion assay [24]. Briefly, polyvinylpyrrolidone-free polycarbonate filters (Millipore, Co. Cork, Ireland) with 12 µm pores were coated with Matrigel (Sigma-Aldrich). Fresh DMEM supplemented with 10% heat-inactivated FBS was placed in the lower chamber as chemoattractant and 1×10^5^ cells were then seeded in the upper chamber and incubated for 6 hrs with gabexate mesilate and cetuximab, alone and in combination, at 37°C in humidified 5% CO_2_. At the end of incubation, non invading cells were removed from the upper surface of the filters. Invading cells in the lower surface were then fixed for 1 min in ethanol 95% and stained for 10 min with 0.5% w/v toluidine blue. In the migration assay, the same procedure of invasion assay was followed, except that filters were coated with gelatin (Sigma-Aldrich). Photographs were taken with an Olympus CKX41 inverted microscope (Olympus Italia, Milan, Italy), equipped with an Olympus C5060-ADU camera (Olympus Italia). For each sample, four random optical fields at ×200 of total magnification was analyzed and the mean number of invading cells was calculated as follows: mean number of invading cells/mean number of corresponding migrating cells.

### In vitro Angiogenesis Assay

In vitro angiogenesis was assessed by endothelial cell differentiation in capillary-like structures in Matrigel. Briefly, growth-factor-enriched Matrigel (BD Biosciences, Bedford, MA, USA) was placed in an ice-cold 24-well plate and left to polymerize for 1 h at 37°C. EA.hy926 endothelial cells (1.2×10^5^/well) were then plated and incubated for 24 hrs with an equal amount of CACO-2, SW48, HT-29, Colo205, SW480, SW620, RKO, LS174T and HCT-116 conditioned medium, obtained as described above in the Material and Methods section. Cancer cell conditioned medium is indeed commonly used in in vitro angiogenesis assays to mimic what occurs during in vivo tumor angiogenesis [25]. At the end of incubation, a three-dimensional organization was observed through an inverted phase-contrast light microscope. Photographs of four fields representative of each sample were obtained at ×200 total magnification with an Olympus CKX41 inverted microscope (Olympus Italia), equipped with an Olympus C5060-ADU camera (Olympus Italia). A semi-quantitative measurement of capillary-like structures (tube formation index) was performed as previously described [26].

### Statistical Analysis

Results are expressed as percentage mean ±S.D. Data were analysed by ANOVA followed by Bonferroni’s post hoc test, using PRISM 5.01 software version (GraphPad Software, San Diego, CA, USA). A p value <0.05 was considered statistically significant.
